# P-1870. Clinical Outcomes of Self-Administered Outpatient Parenteral Antimicrobial Therapy over 12 Years in a Safety Net Hospital

**DOI:** 10.1093/ofid/ofae631.2031

**Published:** 2025-01-29

**Authors:** Anna Jacobs, Jillian Smartt, Michael Harms, Laila M Castellino, Kristin Alvarez, Kavita Bhavan

**Affiliations:** UT Southwestern, Dallas, Texas; Parkland Health, Dallas, Texas; Parkland Health, Dallas, Texas; University of Texas Southwestern Medical Center, Dallas, TX; PHHS, Dallas, Texas; Parkland Health, Dallas, Texas

## Abstract

**Background:**

Outpatient Parenteral Antimicrobial Therapy (OPAT) is safe and effective for medically stable patients requiring intravenous (IV) antibiotics.^1^ OPAT is most commonly administered in a healthcare setting (H-OPAT), utilizing home health services, infusion centers, nursing homes, or hemodialysis centers. However, patients without health insurance are often unable to afford H-OPAT services, leading to lengthy hospital stays and potentially avoidable healthcare costs. Self-administered OPAT (S-OPAT) is relatively uncommon. At our safety net hospital, we established a S-OPAT clinic for uninsured patients in 2009. Data published from the first four years of our S-OPAT program in 2015 demonstrated a decreased risk of 30-day readmission among S-OPAT compared to H-OPAT with no significant difference in 1-year mortality.^1.^ A registry of all patients discharged on OPAT has since been developed.

**Table 1:**
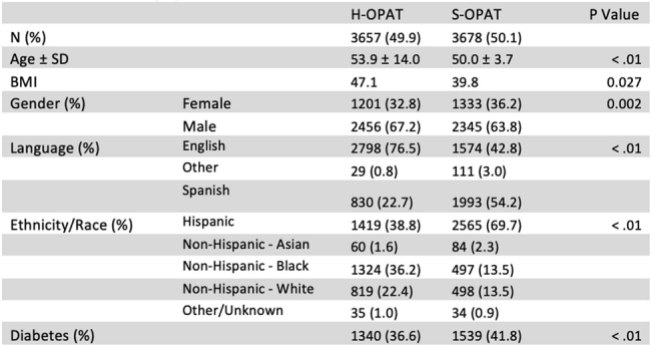
Patient Demographics

**Methods:**

Data from 7335 OPAT discharges between October 2012 and December 2023 were analyzed. We assessed both H-OPAT and S-OPAT groups for blood stream infections secondary to vascular access sites in the ambulatory setting, 30-day readmission rates, and total number of S-OPAT days as an estimate for prevented inpatient days.

**Table 2.**
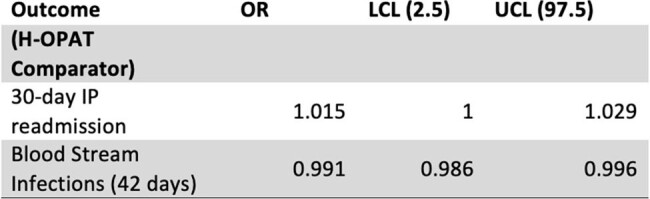
Clinical Outcomes for S-OPAT versus H-OPAT

**Results:**

Of the 7,335 OPAT discharges from 2012 to 2023, 49.9% were discharged to H-OPAT and 50.1% to S-OPAT (Table 1). There was no difference between 30-day readmissions (OR 1.01 [CI 1.000 - 1.029) for S-OPAT as compared to H-OPAT (Table 2). There were a total of 47 blood stream infections over this 12-year period. 35 occurred in H-OPAT patients and 12 in S-OPAT patients (p < .01). A total of 54,733 days of S-OPAT were prescribed, preventing an estimated same number of inpatient days.

**Conclusion:**

S-OPAT was designed to provide an alternative to prolonged hospital stays for uninsured patients requiring prolonged IV antibiotics. There is always a concern about the safety of discharging patients with vascular access, especially in patients with limited access to healthcare. Our data demonstrates that the safety of S-OPAT is as good, if not better, than the standard of care, H-OPAT, while allowing for more effective utilization of and improving health equity for our uninsured patients in our safety net hospital.

**Disclosures:**

All Authors: No reported disclosures

